# Post-Translational Regulations of Foxp3 in Treg Cells and Their Therapeutic Applications

**DOI:** 10.3389/fimmu.2021.626172

**Published:** 2021-04-12

**Authors:** Yi Dong, Cuiping Yang, Fan Pan

**Affiliations:** ^1^ Department of Cell Biology, Johns Hopkins University School of Medicine, Baltimore, MD, United States; ^2^ Department of Gastroenterology, Ruijin Hospital, Shanghai Jiaotong University School of Medicine, Shanghai, China; ^3^ Institute of Biomedicine and Biotechnology, Shenzhen Institute of Advanced Technology, Chinese Academy of Science, Shenzhen, China

**Keywords:** regulatory T cells, Foxp3, post-translational regulation, ubiquitination, glycosylation, acetylation, phosphorylation, therapeutic application

## Abstract

Regulatory T (Treg) cells are indispensable for immune homeostasis due to their roles in peripheral tolerance. As the master transcription factor of Treg cells, Forkhead box P3 (Foxp3) strongly regulates Treg function and plasticity. Because of this, considerable research efforts have been directed at elucidating the mechanisms controlling Foxp3 and its co-regulators. Such work is not only advancing our understanding on Treg cell biology, but also uncovering novel targets for clinical manipulation in autoimmune diseases, organ transplantation, and tumor therapies. Recently, many studies have explored the post-translational regulation of Foxp3, which have shown that acetylation, phosphorylation, glycosylation, methylation, and ubiquitination are important for determining Foxp3 function and plasticity. Additionally, some of these targets have been implicated to have great therapeutic values. In this review, we will discuss emerging evidence of post-translational regulations on Foxp3 in Treg cells and their exciting therapeutic applications.

## Introduction

Thanks to the precious gift inherited from jawed fish ancestors thought to have lived some 500 million years ago, our immune defenses are equipped with a powerful adaptive arm with the ability to mount responses to a near-infinite diversity of targets (1). However, these immune responses can sometimes be overpowered or misdirected to target self-targets. Fortunately, complex mechanisms of immune tolerance have co-evolved to ensure proper regulations on both adaptive and innate immune systems—an immune homeostasis state in the absence of an imminent threat. While central tolerance prevents the development of self-reactive T lymphocytes during their maturation in the thymus, mechanisms of peripheral tolerance suppress the activation of rogue T cells that escape this safeguard (2). Among these, regulatory T (Treg) cells play essential roles in maintaining immune homeostasis by moderating the intensity of immune responses and suppressing the activation of self-reactive leukocytes (3).

The concept of a regulatory T cell lineage has its roots in the “suppressor T cells”. Back in 1969 and 1970, Nishizuka and Gershon found that certain subsets of T cells from the thymus had the ability to suppress the activity of other immune cells (4, 5). Later, in 1995, Sakaguchi identified CD25 (IL-2 receptor α chain) as a reliable marker for these CD4^+^ suppressive T cells (6). This minor T cell population constitutes 5–10% of the peripheral CD4^+^ T cell pool in normal naïve adult mice (i.e., those kept in specific pathogen free (SPF) condition without pathogen challenge), and loss of these cells led to severe autoimmune diseases (7). In 2001, the mutation of a gene encoding the transcription factor Forkhead box P3 (Foxp3) was identified as the disease-causative event underlying IPEX (Immune dysregulation, Polyendocrinopathy, Enteropathy, X-linked syndrome) syndrome in humans and the *Scurfy* phenotype in mice (8–10). Immediately after the discovery of Foxp3, in 2003, Sakaguchi, Rudensky, and Ramsdell confirmed that Foxp3 is the master transcription factor that programs the development and function of regulatory T cells (11–13). Since then, intensive research has been focused on Treg cells to elucidate their roles in different tissues and under a variety of physiological and pathological conditions. It is now well-appreciated that Treg cells play conserved functions in tissue pathophysiology and metabolism across vertebrate species. In addition to their more classical role in immune suppression, these cells are shown to participate in wound healing, tissue homeostasis, and regeneration (14–16). More detailed reviews in this perspective can be found elsewhere (17–19).

After decades of studies, we now understand that these Foxp3^+^ Treg cells use multiple different mechanisms to suppress immune activation. These include mechanisms acting through direct cell-cell interactions. For example, Treg cells express coinhibitory molecules like LAG3, CTLA-4, GITR, and PD-1 to inhibit DC maturation (20–23) and effector T cell function (24). Paracrine release of suppressive mediators is another avenue for suppression. Treg cells are known to produce anti-inflammatory cytokines like TGF-β, IL-10, and IL-35 to inhibit effector T cells. Indirect means have also been studied, and Treg cells are known to sequester the growth factor IL-2 from other leukocytes, thus disrupting their survival and function (25). Losing or disrupting Foxp3 undercuts these diverse mechanisms of immune suppression significantly, which leads to a loss of immune control and severe autoimmune diseases (25).

So far, multiple layers of regulations have been identified to control the function and the turnover of Foxp3, especially transcriptional and post-translational regulations (26, 27). Among these, post-translational regulation is of high interest due to its versatility and specificity. This is because each of these modifications is orchestrated by a highly specific set of proteins with a variety of functions. For example, ubiquitin-dependent modification involves many unique E3 ligases for either poly-ubiquitination-dependent proteasome degradation or monoubiquitinated modification for signaling cascades (28). Due to these features, post-translational regulation provides new insights into protein functions and therapeutic targets (29).

Since the post-translational regulation of Foxp3 has become, of late, an expansive topic, in this review, we will focus on a selective number of modifications, including phosphorylation, dephosphorylation, acetylation, deacetylation, methylation, glycosylation, ubiquitination, deubiquitination, and others. Among these modifications, we will put additional emphasis on ubiquitination and deubiquitination, and discuss some recent therapeutic applications of Treg cells to build a connection between basic research and clinical transformation.

## Classification of TREG

After decades of studies, we now understand that Foxp3^+^ Treg cells are composed of a heterogeneous pool of cells arising from distinct tissues of origin (30). Treg subsets have been characterized with different potentials for proliferation and suppression due to the interplay of both intrinsic and extrinsic mediators (31). Accordingly, many different criteria have been suggested to stratify and classify Tregs. Here, we will only cover some of the most general approaches.

Based on their origin, Tregs can be classified as those having developed in the thymus or those induced in peripheral tissues. The former is normally called natural Treg (nTreg) or thymic Treg (tTreg) and the latter is known as peripheral Treg (pTreg) or inducible Treg (iTreg), especially when generated *in vitro*. nTreg cells are developed from CD4^+^CD8^-^ single positive T cells in the thymus, and iTreg cells are derived from non-Treg precursors (i.e., naïve CD4+ T cells or CD4^+^ T conv cells) (32). While both nTreg and iTreg require IL-2 to maintain cell survival and suppressive function (33–36), TGF-β is critical for iTreg induction (32, 37). Additionally, IL-35, retinoic acid, strong TCR activation (signal 1) with weak co-stimulation (signal 2), and commensal microbiota may also be important for iTreg induction and development (38–46). Last but not least, the TCRs of nTregs are thought to generally recognize self-antigens (47, 48), while iTregs tend to express TCR targeting foreign antigens (49–51). Despite some controversies (52–58), a common approach to distinguish tTreg and pTreg is based on transcription factor Helios and cell surface glycoprotein neuropilin-1 (NRP-1), which are highly expressed on nTreg cells (59, 60).

Treg cells can also be classified by different means, including functional and phenotypic distinctions (61). For example, in terms of suppressive function and proliferation potential, human Treg cells can be classified as CD45RA^+^Foxp3^int^ resting Treg (rTreg) or CD45RA^-^Foxp3^high^ effector Treg (eTreg) cells. Although both rTreg and eTreg are immunosuppressive *in vitro*, rTreg cells have a lower suppressive activity than eTreg cells. However, rTreg cells can be activated to proliferate and differentiate into eTreg cells, but eTreg cells have been described as anergic and prone to apoptosis (31). Furthermore, reflecting distinct activation states, Tregs can be classified as Foxp3^pos^Helios^neg^ or Foxp3^pos^Helios^pos^ Treg cells. The latter represents recently activated Tregs as they express higher Foxp3 and significantly more Ki67 (62). Overall, Treg cells are highly heterogenous and proper criteria are needed to further elucidate their differences.

## Foxp3 Structure and Its Co-Regulators

Foxp3 belongs to the forkhead box (Fox) family, subfamily P. In mammals, there are four members in this subfamily, namely Foxp1–4 (63). Among these, FOXP3 is well conserved among mammals. For example, human and mouse FOXP3 share 91% similarity in amino acid sequences (64). In human, FOXP3 gene locates on the short arm of Chromosome X (Xp11.23). It is about 20,039 bp long including 11 coding exons and one non-coding exon (exon 1) (10). In terms of Foxp3 transcription, four alternatively spliced isoforms have been identified, including full-length Foxp3 (1,869 bp), Foxp3△2, Foxp3△7, and Foxp3△2△7. Among these, 70% of Foxp3 mRNAs are Foxp3△2, which lack retinoic acid-related orphan receptor γt (RORγt) interaction domain (65). Besides, both full-length Foxp3 and Foxp3△2 produce Foxp3 protein with normal functions (66).

**Figure 1:**
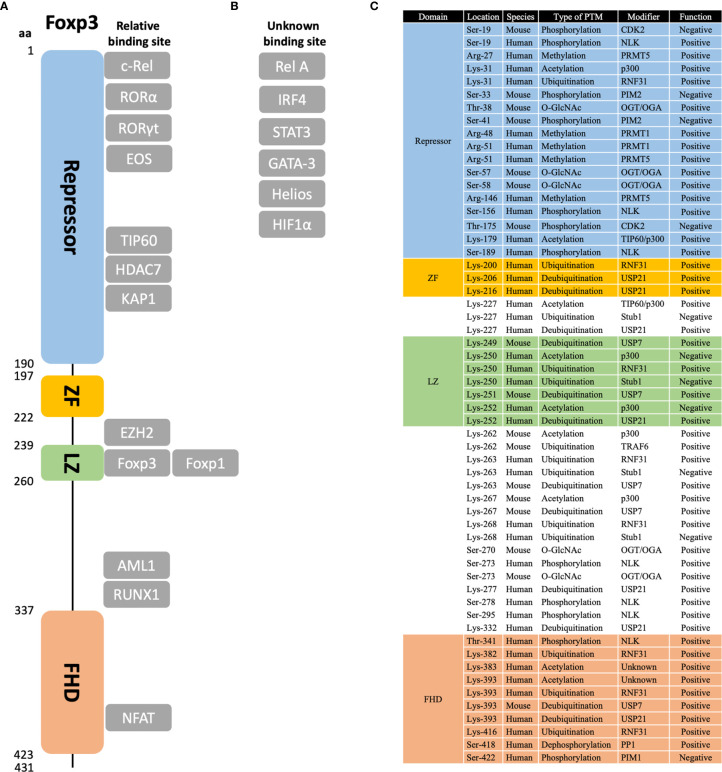
Protein domains, binding partners, and post-translational modification sites of Foxp3. **(A)** The relative structure of Foxp3 protein is shown in scales on the left. Four main domains are marked in different colors, namely repressor domain, zinc-finger (ZF) domain, leucine zipper (LZ) domain, and Fork-head (FHD) domain. **(B)** Main binding partners of Foxp3 are shown in the figure. Those with known binding sites are shown in their relative positions; those with unknown binding sites are listed in “unknown binding site”. **(C)** For post-translational modifications (PTM) of Foxp3, the sites that are discussed in this review are listed in amino acid order. Besides, the species that are examined, the type of PTM, the modifier protein, and the role in regulating Foxp3 function are listed. Note that only those well-characterized sites are listed. Potential or uncertain sites are not included.

Foxp3 protein is about 431 amino acids long and 47.24 kDa in molecular weight. It includes four functional domains, namely the repressor (aa 1–190), zinc finger (ZF, aa 197–222), leucine zipper (LZ, aa 239–260), and Fork-head domain (FHD, aa 337–423) (67) (**Figure 1A**). In brief, the repressor domain is located at the N-terminal region and is required to suppress NFAT-mediated transcriptional signaling, for example, suppressing IL-2 production (68). The zinc finger domain is necessary and sufficient for Foxp3 homodimerization and heterodimerization with Foxp1 (69), thus regulating its transcriptional regulatory functions (70). Similarly, the leucine zipper domain also mediates Foxp3 oligomerization by forming a dynamic two-strand anti-parallel α-helical coiled-coil structure (70). Finally, the Forkhead domain is important for DNA binding and nuclear import (71). It recognizes a core 7 bp DNA-binding sequence 5’-RWAAAYA-3’ (R = A/G; W = T/A; Y = C/T) (72–74).

As the master transcription factor of Treg cells, Foxp3 can form large protein complexes with other co-factors. These complexes vary from 300 to 1,200 kDa in size and may involve up to 361 different potential partners (75, 76). Some of the characterized co-factors are: NFAT (77), RUNX1 (78), RORα (79), Rel A and c-Rel (80), IRF4 (81), Eos (82), STAT3 (83), HIF1α (84), GATA-3 (75), KAP1 (85), EZH2 (86), and Helios (87) (**Figure 1B**). Besides, these co-factors can further bind with other factors to form distinct functional complexes, including transcriptional activation/repression, ubiquitination, acetylation, etc. (76).

## Regulation of Foxp3

Foxp3 is tightly regulated by a network of different mechanisms with certain redundancy. Epigenetically, Foxp3 can be regulated DNA methylation, histone modification, and nucleosome positioning (88). For example, Foxp3 expression can be regulated by the conserved non-coding sequences 2 (CNS 2) within the Foxp3 locus through DNA methylation (89). Transcriptionally, USP22 leads to H2BK120Ub on chromatins among the FOXP3 locus to enhance its transcription (90). As for post-transcriptional modifications, many factors play important roles in regulating the conversion of precursor Foxp3 messenger RNA transcripts into mature messenger RNA. For example, microRNAs (miRNAs) including miR-24, miR-31, and miR-210 can lead to Foxp3 mRNA degradation, which prevents Foxp3 translation (91, 92). In this review, we will briefly cover transcriptional regulation of Foxp3 and specifically discuss post-translational regulation of Foxp3.

### Modification of Foxp3 Expression at Transcriptional Level

Foxp3 can be regulated by a number of cis-acting elements, which are located on the promoter and the enhancer regions (CNS0, CNS1, CNS2, and CNS3) of the Foxp3 locus (93, 94). These regions contain binding sequences for transcription factors that are induced by extracellular signaling, including TCR, CD28, TGF-βR, and IL-2R signaling (89). In this section, we will review four major pathways that regulate Foxp3 at the transcriptional level.

Upon T cell stimulation, TCR-induced NF-κB pathway plays an important role in regulating Foxp3 expression and Treg development. Mutating or deleting many key enzymes within the pathway, including PKC-θ (95), Bcl-10 (96), CARMA-1 (97, 98), IKK2 (99), and c-Rel (100–102), have been shown to reduce Foxp3 expression and Treg frequency significantly. Further studies have shown that c-Rel is the key NF-κB subunit that binds to the promoter, CNS2, and CNS3 to regulate Foxp3 transcription (93, 103).

Another important pathway is the PLCγ–NFAT/AP-1 pathway. Upon TCR engagement, PLCγ induces calcium influx, which leads to the activation of NFAT1 (104). NFAT1 not only plays an important role in maintaining Foxp3 expression (105), but also acts collaboratively with Foxp3 to regulate IL-2, CTLA-4, and CD25 (77). Besides, PLCγ induces the activation of FOS/JUN, which leads to the activation of the AP1-NFAT transcriptional complex (106). In the Foxp3 locus, three NFAT and three AP1 binding sites are situated in close proximity to each other, which further supports their collective role in regulating Foxp3 expression (107).

Besides T cell stimulation, IL-2 signaling is also important for Treg survival and function. It has been shown that IL-2 receptor subunits IL-2Rβ (CD122) and IL-2Rγ (CD132) are essential for Foxp3 expression, as losing them leads to no detectable Foxp3^+^ T cells in mice (108–110). Additionally, losing IL-2Ra would impair the development of Treg cells in the thymus and reduce the suppressive function in the peripheral (111, 112). In brief, upon IL-2 binding, the IL-2 receptor triggers JAK1 and JAK3 phosphorylation, which leads to the activation of STAT3 and STAT5 (113). Then, dimerized STAT5 will bind to the promoter and enhancer in CNS2 region, supporting the transcription of Foxp3 (110, 114). This signaling pathway is important for Treg development in the thymus (110, 114). It also plays a major role in the homeostasis and function of Treg cells in the peripheral (115, 116).

TGF-β is an important cytokine for both Treg induction and maintenance (33). Recent studies have explained the role of this TGF-β-Smad signaling in regulating Foxp3 expression. Both Smad2 and Smad3 are redundantly essential in regulating Foxp3 expression, demonstrated by double knockout mouse models (117). In addition, Smad2/3 binds to the Foxp3 locus in a temporal and spatial order. In brief, it first binds to the enhancer region of CNS1 and then dissociates from it in order to bind to the promoter binding element, which is about −85 bp upstream of the transcription start site (105, 118). Overall, Foxp3 is regulated by many important factors at the transcriptional level.

### Modification of Foxp3 Expression and Function at Post-Translational Level

**Figure 2:**
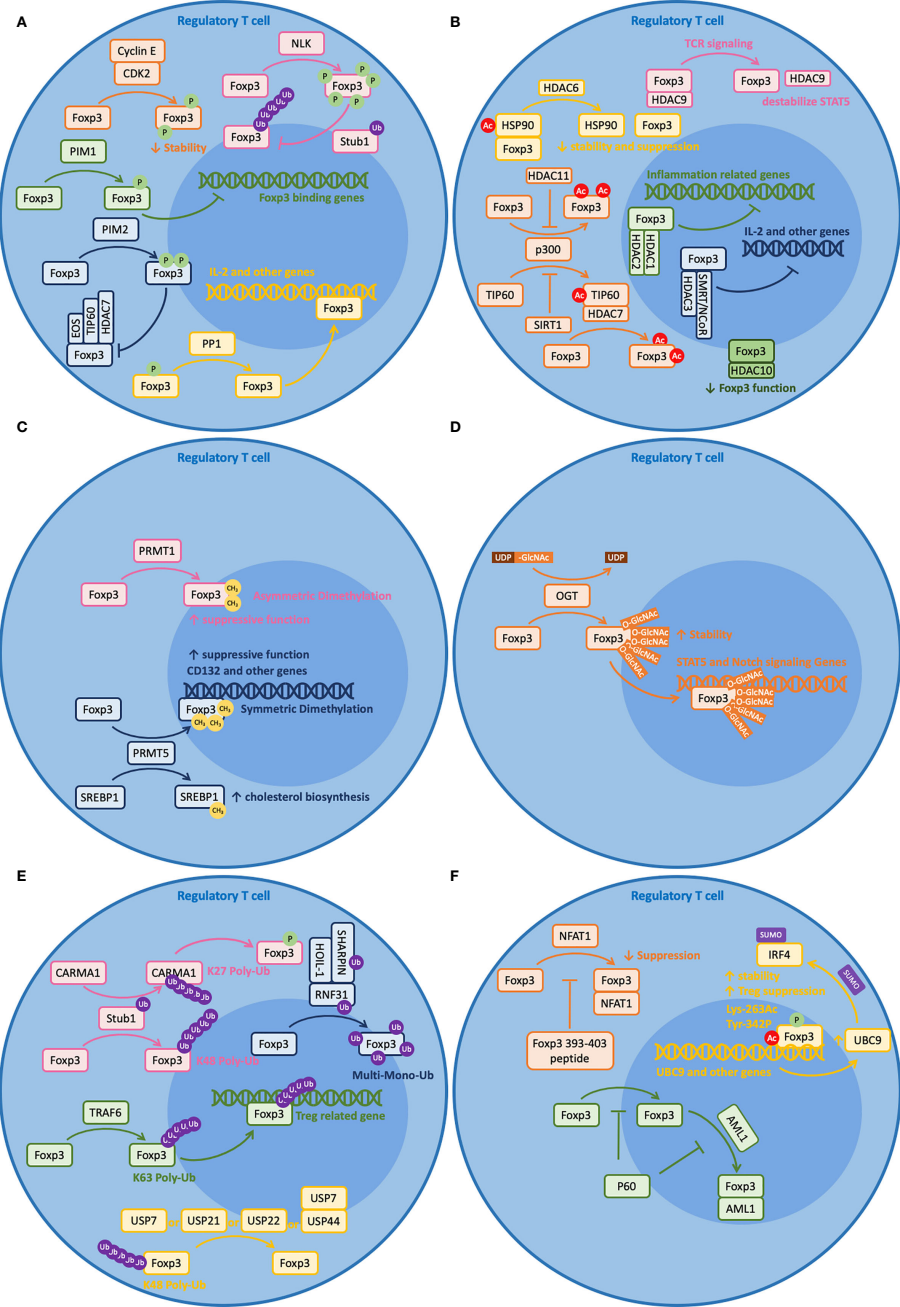
Post-translational modifications (PTM) of Foxp3. Foxp3 can be post-transcriptionally modified by a variety of regulators. Brief illustrations are shown here to outline their basic mechanisms. **(A)** Phosphorylation (CDK2, PIM1, PIM2, NLK) and dephosphorylation (PP1) of Foxp3. **(B)** Acetylation (p300 and TIP60) and deacetylation (HDAC1, 2, 3, 6, 7, 9, 10, 11, and SIRT1) of Foxp3. **(C)** Methylation (PRMT1 and PRMT5) of Foxp3. **(D)** Glycosylation (OGT) of Foxp3. **(E)** Ubiquitination (RNF31, Stub1, and TRAF6) and deubiquitination (USP7, 21, 22, and 44) of Foxp3. **(F)** Other modifications, including peptides (Foxp3 393–403 peptide and P60) that regulate Foxp3 and SUMOlyation (UBC9) that is regulated by PTM modified Foxp3.

Post-translational modification (PTM) refers to enzymatic processes that alter a protein after its synthesis. These modifications will influence the characteristics of the protein, including its localization, interaction, turnover, etc. Given the importance of this factor and the T cell lineage it defines, Foxp3 is, not surprisingly, tightly regulated by a number of mechanisms involving post-translational modifications. In this section, we will review some recent findings on post-translational modifications of Foxp3 (**Figure 1C**) and their roles in maintaining Foxp3 function and plasticity (**Figure 2**).

#### Phosphorylation and Dephosphorylation

Phosphorylation is a mechanism by which a protein kinase attaches a covalently bound phosphate group to the serine (S), threonine (T), or tyrosine (Y) residue of a protein. It is a reversible process as the protein can be dephosphorylated by phosphatases. These two modifications are important for protein function and stability. For Foxp3, more than 15 sites have been documented to be targets of phosphorylation. Among these, kinases CDK2, PIM1, and PIM2 negatively regulate Foxp3 functions; kinase NLK and phosphatase PP1 positively regulate Foxp3 function (119) (**Figure 2A**).

CDK2, PIM1, and PIM2 suppress Foxp3 function through different mechanisms. CDK2 is known to reduce Foxp3 stability. Along with cyclin E, CDK2 binds to four cyclin-dependent kinase motifs within the N-terminal domain of Foxp3 and causes phosphorylation of at least Ser-19 and Thr-175 (120). Such phosphorylation events lead to reduced Foxp3 protein stability and reduced Treg suppressive function (121). Intriguingly, PIM1 does not reduce Foxp3 stability but affects its DNA binding activity. Under inflammatory conditions, PIM1 phosphorylates Foxp3 at Ser-422 directly, which is located at the C-terminal region of the Fork-head domain (FHD) (122). Since the Fork-head domain is important for DNA binding, phosphorylated Ser-422 leads to reduced DNA binding. Likewise, PIM2 does not reduce Foxp3 stability but may affect its protein binding activity with other cofactors. PIM2 phosphorylates the N-terminal region of Foxp3, including at least Ser-33 and Ser-41 (123). These regions are important for binding with other cofactors like Eos, TIP60, and HDAC7, so phosphorylation at these sites may affect their binding activities (123, 124).

NLK and PP1 promote Foxp3 function through different mechanisms. NLK improves Foxp3 stability by preventing ubiquitin-dependent protein degradation of Foxp3. Upon TCR stimulation, TAK1–NLK signaling is activated (125). Then, NLK phosphorylates Foxp3 at seven distinct sites: Ser-19, Ser-156, Ser-189, Ser-273, Ser-278, Ser-295, and Thr-341 (126). Such phosphorylation prevents its interaction with STUB1, an E3 ligase that induces K-48 polyubiquitination of Foxp3 (126). As for PP1, it protects Foxp3 function by improving DNA binding. Under inflammatory states, TNF-α induces an unknown kinase that phosphorylates Foxp3 at Ser-418 (127). Since Ser-418 resides in the Forkhead domain (FHD) and is critical for IL-2 regulation, dephosphorylation at Ser-418 by PP1 protects the suppressive activity of Foxp3 (127).

#### Acetylation and Deacetylation

Acetylation is both a co-translational and a post-translational modification procuress that introduces an acetyl group into a protein by histone acetyltransferases (HTAs). This process can be reversed by histone deacetylases (HDACs). In regulatory T cells, acetylation and deacetylation of Foxp3 protein are mostly regulated by three HATs and nine HDACs with certain redundancy (128) (**Figure 2B**).

TIP60, p300, and CBP are the three HATs that regulate Foxp3 acetylation. Among these, CBP is a paralog of p300 and they perform critical but relatively redundant functions on Foxp3, based on double knockout experiments (129). Experiments have shown that p300 acetylates Foxp3 at multiples sites, including at least Lys-31, Lys-262, and Lys-267. These sites are critical for Foxp3 stability as they are also sites served for ubiquitin-dependent protein degradation (130). TIP60 regulates Foxp3 acetylation through multiple mechanisms. Firstly, it can form a binding complex with HDAC7 to promote Foxp3 acetylation and target the 106–190 aa repressor domain near the N-terminal region of Foxp3, which is important for IL-2 repression (131). Additionally, it can work cooperatively with p300 to enhance Foxp3 acetylation. It has been shown that p300 can facilitate the autoacetylation of TIP60 at Lys-327, allowing TIP60 to change binding partners to further enhance Foxp3 acetylation (132). Together, optimized Foxp3 acetylation shall be achieved in the presence of both TIP60 and p300 (for example, at Lys-179 and Lys-227) (133). Experiments have shown that losing either one of them leads to weak Foxp3 acetylation (132).

Besides direct acetylation by TIP60, p300, and CBP, it has been shown that Foxp3 structure can influence its interaction with HATs and vice versa. A recent study has shown that p.A384T FKH mutation on Foxp3 can disrupt the interaction between Foxp3 and TIP60, impairing the development and suppressive function of Treg cells (134). On the other hand, acetylation of Lys-250 and Lys-252 on Foxp3 by p300 leads to dimer relaxation and downregulates the suppressive function of Foxp3 (135). What is more, TGFβ induced Foxp3 acetylation and DNA binding are regulated by unknown mechanisms at Lys-383 and Lys-393 (136, 137). Taken together, these demonstrate the importance of protein acetylation on Foxp3.

HDACs from five different subfamilies are involved in Foxp3 deacetylation, including HDAC1, 2, 3 from Class I, HDAC6, 7, 9 from Class IIA, HDAC10 from Class IIB, SIRT1 from Class III, and HDAC11 from Class IV. Among these, Class I HDACs are ubiquitously expressed by all cells. Experiments have shown that HDAC1 and HDAC2 bind to the N-terminal region of Foxp3 and counteract the hyper-acetylation of inflammation-related genes (69). HDAC3 interacts with SMRT/NCoR to form a functional complex in Treg cells. It associates with Foxp3 for optimal suppressive function, including suppressing IL-2 production (138). Class IIa HDACs have a restricted tissue distribution and they are expressed in lymphocytes. Upon inflammatory conditions, HDAC6 deacetylates both Foxp3 and HSP90, preventing them from forming a complex (139). The direct role of HDAC6 in regulating Foxp3 protein interaction and function is yet unclear, but recent experiments have shown that deacetylation of HSP90 affects many client proteins, including HSF1. Additionally, deletion of HDAC6 in Treg cells would increase the expression of many Treg-associated genes, including IL-10, Lag3, STAT3, and decrease IL-2, which significantly improves Treg stability and suppressive function (139, 140). Besides the role of forming functional complex with TIP60 and Foxp3 (131), the HDAC7 complex is also associated with the repression of Nur77, which regulates the balance between Teff and Treg cells and the stability of Treg cells (141). Despite the direct role of deacetylating Foxp3, upon TCR-induced cell signaling, HDAC9 dissociates from Foxp3 and disrupts the acetylation of STAT5, which negatively regulates Foxp3 function (131, 136, 142). As a Class IIB HDAC, HDAC10 also interacts with Foxp3 directly and reduces the suppressive function of Foxp3. *In vitro* experiments show that HDAC10 promotes the deacetylation of Foxp3 at Lys-31. However, the detailed mechanism remains elusive (143). Different from the other HDACs, SIRT1 is NAD-dependent, and it inhibits the autoacetylation of TIP60, which negatively regulates the acetylation of Foxp3 (144, 145). Lastly, as the only member of Class IV Zn^2+^-dependent HDAC, HDAC11 co-associates with Foxp3 in the nucleus and promotes p300-dependent deacetylation of Foxp3. Besides, it has been reported to regulate the suppressive function of Foxp3 *in vitro* through a TGF-β-dependent mechanism by regulating the expression of Treg-associated genes (146).

#### Methylation

Protein methylation is a common type of post-transcriptional modification that adds methyl groups to a protein, commonly on arginine and lysine residues (147). Recent studies have reported that two arginine methyltransferase (PRMT) family members, PRMT1 and PRMT5, are critical for Foxp3 protein methylation (119) (**Figure 2C**).

In detail, PRMT1 leads to asymmetrical dimethylarginines on Arg-48 and Arg-51 of Foxp3 (148). Inhibiting the methylation of these two sites simultaneously leads to reduced suppressive function of Treg cells and increased Th1-associated gene expression profiles in Foxp3^+^ T cells (148). Using MS023 [a type 1 protein arginine methyltransferase (PRMT) inhibitor]-treated WT Foxp3+ T cells and Foxp3 R48/51A-transduced T cells, these authors suggested that repressing the Th1 phenotype through an AKT-dependent signaling abrogates the effect of arginine methylation inhibition on the suppressive activity of Foxp3+ T cells (148). Interestingly, another study showed that PRMT1 is also important for differentiation of Th17 cells by associating with RORγt and regulating the reciprocal recruitment of STAT3 and STAT5. These authors showed that overexpression of PRMT1 promotes Th17 differentiation and inhibition of PRMT1 expands Foxp3^+^ Treg cells population (149). Taken together, these two studies reveal the role of PRMT1 in regulating Foxp3 function and Treg plasticity.

Likewise, PRMT5 catalyzes symmetric dimethylarginines on Arg-27, Arg-51 and Arg-146 of Foxp3 (150), which are confirmed using point mutation experiment and mass spectrometry. Among these, methylation on Arg-51 is critical for the suppressive function of Foxp3. Conditionally knocking out PRMT5 in mouse Foxp3^+^ cells lead to reduced numbers of Treg cells in the spleen but not in peripheral lymph nodes (150). In addition, silencing PRMT5 in these Foxp3+ cells resulted in limited suppressive function, demonstrating the importance of PRMT5 in maintaining Treg functions. Additionally, human CD4+T cells transfected with Foxp3 R51K, a mutant lacking di-methylation site modified by PRMT5 showed reduced suppressive functions compared to those with vector encoding wild type Foxp3 (150). A more recent study demonstrated that PRMT5 is not required for thymic development of T cells before the double positive (DP) stage (151). However, the factor is critical for peripheral T cell survival, naïve to effector/memory transition, and TCR-induced proliferation. One of the potential mechanisms responsible for these observations involves RPMT5 regulation of the expression of the common gamma chain (CD132) of IL-2 receptor (151). Besides, PRMT5 modulates Th17 differentiation *via* the methylation of SREBP1, which regulates cholesterol biosynthesis (152). Taken together, both PRMT5 and PRMT1 are critical in regulating Foxp3 function and plasticity.

#### Glycosylation

Glycosylation is a form of co-translational and post-translational modifications that attaches glycans to proteins (153). As a rising field in biological studies, protein glycosylation has shown important effects on T cell development, activation, and differentiation (153). Some studies have shown that the surface glycosylation patterns of regulatory T and conventional T cells are very different (154, 155). Interestingly, surface levels of tri/tetra-antennary N-glycans on Treg cells correlates with the expression level of suppressive ligands, including GITR, PD-1, PD-L1, CD73, CTLA-4, and ICOS. Further experiments have suggested a positive correlation between glycosylation and the suppressive activity of Treg cells (154).

Recently, it has been shown that Foxp3 can be modified by TCR-activated O-linked N-Acetylglucosamine (O-GlcNAc) at multiple sites, including Thr-38, Ser-57, Ser-58, Ser-270, and Ser-273 (156). Using Treg cells from inducible O-GlcNAc transferase (OGT) knockout mice (Ubc-Cre/ERT2^+^Ogt^fl/Y^), these authors showed that loss of O-GlcNAcylation destabilizes Foxp3 protein in Treg cells *ex vivo*. Furthermore, using liquid chromatography with tandem mass spectrometry (LC-MS/MS) through electron transfer dissociation (ETD), these authors further found the above O-GcNAc sites using HEK293 cells expressing Foxp3. Although *O*-GlcNAc-deficient Treg cells develop normally in mice (Foxp3^YFP-Cre/Y^Ogt^fl/Y^), their Foxp3 expression level is moderately decreased and their suppressive function is significantly impacted partially due to attenuated IL-2/STAT5 and Notch signaling (156, 157). Besides the role of O-GlcNAc on Foxp3, it also affects the activation and function of c-Rel, which is a critical NFκB subunit that regulates Foxp3 transcription (158, 159) (**Figure 2D**).

#### Ubiquitination and Deubiquitination

Ubiquitination is a type of post-translational modification that attaches ubiquitin molecules to the target protein (160). It is an ATP-dependent regulation that involves three adapter proteins, namely E1 (ubiquitin-activating enzyme), E2 (ubiquitin-conjugating enzyme), and E3 (ubiquitin ligase). The substrate protein can be either monoubiquitinated or polyubiquitinated. While monoubiquitination normally involves cell signaling and membrane trafficking, polyubiquitination involves many different functions. Among the eight different types of polyubiquitination (K6, K11, K27, K29, K33, K48, K63, and M1), K48 polyubiquitination leads to proteasome-dependent protein degradation and K63 polyubiquitination involves cell signaling (161). Ubiquitination is a reversible process and it can be reversed by deubiquitinases (DUB), which remove ubiquitin tags from the substrate protein (162).

In Treg cells, RNF31, Stub1, and TRAF6 are the three E3 ligases that directly ubiquitinate Foxp3 (**Figure 2E**). Interestingly, they mediate different types of ubiquitination that lead to different functions. For example, RNF31 leads to monoubiquitination on Foxp3. In brief, RNF31 is a RING-type E3 ligase of LUBAC (linear ubiquitin chain assembly complex) (163). It is auto-inhibited before binding with the other two proteins of LUBAC, namely HOIL-1, and SHARPIN (164, 165). The complex regulates linear polyubiquitination and is important for multiple immune pathways, including TCR, BCR, NOD, TLR, and TNFR signaling pathways (166–172). In Treg cells, RNF31 not only regulates TCR signaling but also regulates multiple monoubiquitinations on Foxp3, including Lys-31, Lys-200, Lys-250, Lys-263, Lys-268, Lys-382, Lys-393, and Lys-416 (173). These atypical ubiquitin chains lead to improved Foxp3 protein level and enhanced suppressive functions (173, 174).

Unlike RNF31, which mediates monoubiquitination, Stub1 and TRAF6 regulate polyubiquitination on Foxp3. Stub1 is a U-box type E3 ligase that interacts with HSP70 (175). When challenged with stress signals like LPS and proinflammatory cytokines, Stub1 mediates K48 polyubiquitination on Foxp3 at multiple sites, including at least Lys-227, Lys-250, Lys-263, and Lys-268 (176). Such ubiquitination leads to proteasome-dependent Foxp3 degradation and thus reduced suppressive activity (176). Additionally, Stub1 leads to K27 polyubiquitination on CARMA1 at Lys-689 and Lys-696, which facilitates the activation of the NFκB pathway (177). Distinct from the action of Stub1, which leads to Foxp3 degradation, TRAF6 regulates Foxp3 localization. TRAF6 is a RING-type E3 ligase that mediates inflammation-related signals, including IL1R, TLR, and TNFR superfamily signaling (178). Studies from our group have shown that TRAF6 mediates K63 polyubiquitination on Foxp3 at Lys-262, which does not interfere with other K48 polyubiquitination (179). The mechanism behind this is that TRAF6 regulates the nuclear transport of Foxp3 as deficiency of TRAF6 leads to aberrant accumulation of Foxp3 in the cytoplasm (our unpublished data). Together, these studies illustrate the importance and versatility of E3 ligases in regulating Foxp3 stability and function.

As the Janus side of ubiquitination, deubiquitination also plays important roles in regulating Foxp3 function and plasticity. Among nearly 100 DUBs reported so far (162), USP7, USP21, USP22, and USP44 have shown direct interaction with Foxp3 (**Figure 2E**). USP7 preserves Foxp3 homeostasis by removing K48-type polyubiquitination tags at Lys-249, Lys-251, Lys-263, Lys-267, and Lys-393 (180). Thus, USP7 prevents Foxp3 from ubiquitin-dependent protein degradation and enhances the interaction between Foxp3 and TIP60, which preserves its expression level (180, 181). Similarly, USP21 prevents Foxp3 degradation by deubiquitinating K48-type modifications at residues Lys-206, Lys-216, Lys-227, Lys-252, Lys-277, Lys-332, and Lys-393 (182). The action of USP21 also appears to play a role in a feedback loop with Foxp3 (183, 184). Upon TCR stimulation, Foxp3 activates the transcription of the Usp21 gene. Then, USP21 prevents Foxp3 degradation, which further enhances the transcription of Usp21 and suppresses Th1-like phenotypes (182, 183). Likewise, USP22 interacts with Foxp3 and prevents its degradation (90). Recently, a study from our group showed that USP44 also interacts with Foxp3 and prevents its degradation by deubiquitinating K48-type polyubiquitin chains on Foxp3 (185]. During iTreg differentiation, TGF-β signaling induces USP44 upregulation. Then, USP44 cooperates with USP7 to stabilize and deubiquitinate Foxp3 (185). Together, these DUBs play important roles in maintaining Foxp3 homeostasis and show great therapeutic potentials as drug targets.

#### Others

Besides the natural post-translational modifications reviewed above, Foxp3 binding peptides may function as artificial PTMs and are worth discussing. These binding peptides can be either pieces of the Foxp3 amino acid sequence competing for the binding partners and co-factors of Foxp3, or random peptides that block critical Foxp3 functions. So far, two peptides have been carefully studied regarding their roles in regulating Foxp3 functions. First, Foxp3 393–403 peptide can interact with the RHR domain of NFAT1, disrupting of the Foxp3/NFAT1 interaction—a molecular pairing that is thought to regulate Treg-associated genes. Such inhibition reduces Treg suppression and may be useful for tumor immunotherapies (186). Another 15-mer peptide, named P60, originates from a phage-display peptide screening. P60 can enter the cell, inhibit Foxp3 nuclear transportation, and impedes Foxp3 dimerization and Foxp3/AML1 interaction (186, 187) (**Figure 2F**). These findings suggest good therapeutic potential of artificial post-translational modifications of Foxp3.

In addition to post-translational regulations of Foxp3, modified Foxp3 can play a critical role in regulating other PTMs, for example, SUMOylation. Similar to ubiquitination, SUMOylation tags small ubiquitin-like modifiers (SUMO) to the substrate for multiple functions, including cell signaling and protein turnover (188). As the only E2 conjugating enzyme involved in SUMOylation, UBC9 has been reported recently to play a critical role in maintaining Treg suppression by enhancing IRF4 SUMOylation. Such SUMOylation would prevent IRF4 from proteasome degradation (189, 190). As a transcriptional activator of UBC9, Foxp3 binds to the UBC9 promoter and regulates its activation. Furthermore, de-acetylation/ubiquitination at Lys-263 and dephosphorylation at Tyr-342 of Foxp3 would severely impede UBC9 transcription (189), suggesting an important role of Foxp3 PTM in regulating Foxp3 functions (**Figure 2F**).

## Treg Cells in Clinical Applications

As a rising field in its own right, the therapeutic use and modulation of Treg cells can have major clinical applications in the treatment of autoimmune diseases, transplantation, cancer, as well as colitis and other inflammatory diseases. So far, 1,010 clinical trials involving Treg cells have been documented at the NIH. In addition, hundreds of small molecules drugs, antibodies, and other nanoparticles have been designed to target Treg cells directly or indirectly, indicating the broad range of potential applications involving Treg cells (191). In this section, we will review some of the most recent advances in the clinical manipulation of Tregs and provide some potential therapeutic drugs that may worth further investigations.

### Autoimmune Diseases

In brief, autoimmune diseases occur when the immune system mistakenly targets self. Some common autoimmune diseases include Lupus, Celiac disease, Sjogren’s syndrome, Multiple sclerosis, Polymyalgia rheumatica, Type 1 diabetes (T1D), and rheumatoid arthritis (RA) (192). Of note, deficiency or dysfunction of Treg cells is considered to be the leading cause of these diseases. For example, patients suffered from IPEX syndrome have FOXP3 mutations that leads to a reduction of the number of Foxp3+ Treg cells (10); many RA patients have relatively normal number of Treg cells, but a lot of them show impaired suppression of self-antigens (193).

To resolve these issues, many clinical trials are focusing on augmenting functionally suppressive Treg cells for these patients. One way is to select polyclonal autologous T cells, expand iTreg cells *ex vivo*, and then infuse them back to patients. Many trials have been performed using this approach to treat patients with T1D and lupus, including, NCT02772679, NCT02932826, NCT02704338, and NCT02428309 (194).

### Transplantation

Despite established surgical techniques of organ transplantation, long-term tolerance to an allogeneic organ is still challenging. One current approach to prevent graft vs host disease (GvHD) is to use immunosuppressants to down-regulate normal immune functions, including using anti-proliferative agents, steroids, mTOR inhibitors, and Calcineurin inhibitors (195). However, systemic immunosuppression can lead to many side effects, making patients susceptible to infectious diseases and cancer (196).

For these reasons, chimeric antigen receptor Treg (CAR-Treg) therapy attracts a lot of attentions (197, 198). Since MHC I is critical for immune tolerance and HLA-A2 is highly prevalent in white human donors (199, 200), HLA-A2-specific CAR-Treg (A2-CAR-Treg) was generated and examined in mouse models. Surprisingly, in a skin allograft model, NOD.Rag1^null^.IL2rg^null^ (NRG) mice receiving A2-CAR-Treg cells showed much stronger tolerance induction than those receiving polyclonal nTreg or controls (201). Therefore, antigen-specific Treg therapy cells hold a promising future for patients that require transplantation.

### Cancer

While important for maintaining immune homeostasis, Treg cells are known to play a pathological role in cancer patients by impeding desirable anti-tumor immunity (202). They readily accumulate within tumors and suppress the activity of tumor-reactive cytotoxic immune cells, leading to uncontrolled expansion and migration of malignant cells (203). Therefore, strategies to deplete or inhibit intratumoral Treg cells are being pursued to improve the therapeutic outcome.

As a proven weapon in the anti-cancer arsenal, checkpoint inhibitor immunotherapies are being used to maintain and boost the function of cytotoxic T cells (204). From the clinical perspective, blocking antibodies, including ipilimumab (anti-CTLA-4), nivolumab (anti-PD-1), and pembrolizumab (anti-PD-1) have been widely approved to treat many types of cancers (205). Since the ligands of these checkpoints, including PD-1, CTLA-4, are expressed by Treg cells at high levels (206, 207), blocking these checkpoints are also expected to regulate the plasticity of suppressive function of Treg cells (23, 208–210). So far, many theories have been proposed to explain the therapeutic effects of checkpoint inhibitors on Tregs, and they offer explanations for the distinct effects of checkpoint inhibitors on Treg cells and non-Treg cells. First, the anti-CTLA-4 antibody blocks Treg cells from downregulating the B7 ligands on APCs, which leads to reduced CD28 co-stimulation (211, 212). It also prevents the co-inhibitory CTLA-4: B7 binding on Tconv cells, which promotes the activation of Tconv cells (213, 214). Second, the anti-PD-1 antibody prevents the conversion of TBET+ Th1 cells into Foxp3+ Treg cells *in vivo* (215). Additionally, it prevents the PD-1: PD-L1 binding between tumor-infiltrating lymphocytes and tumor cells, which restores the antitumor immunity (23, 216, 217).

Drugs specifically targeting Foxp3 PTM also represent high potential strategies for combating immune suppression in cancer as they are not likely to affect the function of Tconv cells and CTLs. As reviewed in the post-translational modification sections, preventing the acetylation, DNA binding, or dimerization of Foxp3 are potentially useful approaches to suppress Treg cells. Thus, preclinical studies using USP7 inhibitors (181), p300 inhibitors (218), P60 peptide (187), PMRT5 inhibitors (150), and PROTACs (219) may be promising.

### Colitis and Other Inflammation Associated Diseases

We are challenged by a variety of potential pathogens at all times, including bacteria, viruses, and fungi (220). To maintain tissue homeostasis, Treg cells play an important role in developing T cell memory and prevention of reinfection, especially in gastrointestinal and respiratory systems (221). Impaired Treg function or plasticity can lead to chronic inflammation (222). For example, dysfunctional Treg cells can underlie the development of inflammatory bowel disease (IBD) that can also leads to colitis-associated colon carcinoma (CA-CRC) in the long run (222).

Some factors are known to restrict Treg functions, including chronic exposure to inflammatory cytokines and deprivation of stabilizing factors. Chronic exposure of Tregs to pro-inflammatory cytokines like IL-1β, IL-6, TNFα, which can antagonize Foxp3 expression and skews the gene expression profile to that of Th17 or Th1 (223–225). Reduced levels of IL-2 and TGFβ (116, 226), on the other hand, can adversely affect the survival of Treg cells. Therefore, besides anti-inflammatory drugs which relieve colitis symptoms (227), drugs stabilizing and enhancing Foxp3 function may also be useful, such as HADC6 and HADC9 inhibitors (228).

## Summary and Future Perspective

After decades of intensive studies, our knowledge of Treg cells has improved significantly. With increasing regulatory mechanisms of Foxp3 being brought to light, the potential of manipulating Foxp3 at the post-translational level has increased dramatically. At this stage, we believe two aspects will be important for future drug discovery: first, an *in vivo*, high-throughput target screening assay for agents capable of disrupting the posttranslational means of supporting Foxp3 expression and function and the stronger suppressive potency of Treg cells, and second, a high-throughput and cost-efficient pre-clinical drug examination platform for Treg-based therapies. While the former requires more accurate and efficient CRISPR-based gene-editing techniques to be applied to Treg cells, the latter aspect will benefit from new screening systems. On the one hand, murine disease models are still very different from human cases due to evolutionary heterogeneities (229). On the other hand, macaque models are very expensive, time-consuming, and less quantitative due to the limited sample size (230). Besides, a lot of ethical regulations limited the use of primates (231), making it difficult for high-throughput experiments. In this case, using organoids and microfluidic techniques may be a compromise. Since organoids are derived from human stem cells, they can mimic certain functions of human organs *ex vivo* (232). Moreover, microfluidic devices can provide 3D structures for immune cells, bacteria, viruses, and organoids to contact with each other, which may represent *in situ* interactions (233). Thus, 3D *ex vivo* culture systems showed great promise in drug development.

Another equally important issue is how to improve Treg-specific targeting. Currently, most strategies targeting Treg cells use monoclonal antibodies or ligand-directed toxins to deplete Treg cells systemically or locally (234). However, such strategies do not fit all disease conditions, especially autoimmune diseases and other inflammation-associated diseases. Like other cell-type-specific drugs, a biochemistry-based, bioinformatics-guided designing platform is needed to achieve Treg-specific targeting. To do so, the effective first step is to prevent targeting the isoforms of the target protein. For example, treatment of a class I/II HDAC inhibitor, Trichostain A (TSA), increased the percentage of Treg cells and their suppressive activities (136). A later experiment shows that the main effects were caused by inhibiting the class IIb HDACs (e.g., tubastatin A) (228). Since both HDAC6 and HDAC10 belongs to class IIb HDAC and HDAC6 has been demonstrated to play more important roles in Treg functions *in vivo* (235), a specific inhibitor for HDAC6 may provide the optimum therapeutic outcome in the future. Another important way to achieve Treg specificity is to target proteins with relatively unique functions and pathways. In this regard, E3 ligases would be excellent candidates since there are nearly 500–1,000 of them in human and many of them have unique functions in specific tissues or cell types (236). Moreover, it is tempting to use nanoparticles to achieve Treg specificity. Many recent studies have used specifically designed particles as carriers to present antigens or deliver drugs to Treg cells (237–240), which suggests that small-molecule inhibitors against PTM enzymes can also be used for future drug designs. Above all, we believe strongly that investigating post-translational modifications of Foxp3 will reveal more therapeutic potentials in the future and new techniques will facilitate the drug discovery.

## Author Contributions

All authors listed have made a substantial, direct, and intellectual contribution to the work and approved it for publication.

## Funding

This work was supported by the start-up package from SIAT, no. 1105161001.

## Conflict of Interest

The authors declare that the research was conducted in the absence of any commercial or financial relationships that could be construed as a potential conflict of interest.
